# Phytochemical Screening and Antidiabetic, Antihyperlipidemic, and Antioxidant Properties of* Anthyllis henoniana* (Coss.) Flowers Extracts in an Alloxan-Induced Rats Model of Diabetes

**DOI:** 10.1155/2018/8516302

**Published:** 2018-06-24

**Authors:** Ameur Ben Younes, Maryem Ben Salem, Hanen El Abed, Raoudha Jarraya

**Affiliations:** ^1^Department of Chemistry, Laboratory of Organic Chemistry (LR17ES08), Chemistry of Natural Substances Team, Faculty of Sciences, BP 1171, 3000 Sfax, Tunisia; ^2^Laboratory of Pharmacology, Faculty of Medicine, University of Sfax, 3000 Sfax, Tunisia; ^3^Department of Biology, Laboratory of Vegetal Biotechnology Applied in Agriculture Improvement, Faculty of Sciences, University of Sfax, 3000 Sfax, Tunisia

## Abstract

**Background:**

This study investigates the biological activities of* Anthyllis henoniana* flowers extracts.

**Materials and Methods:**

Antioxidant activity and the* in vitro* inhibitory effect of key digesting enzymes related to postprandial hyperglycemia were determined. Diabetic rats were orally and daily given the best extract from flowers of* Anthyllis henoniana* at a dose of acarbose for one month.

**Results:**

Among the extracts, the ethyl acetate one displayed remarkable antioxidant activity including DPPH (IC_50_ = 2.34 mg/mL) and was more effective in inhibiting *α*-glucosidase (IC_50_ = 17 *μ*g/mL) than *α*-amylase (IC_50_ = 920 *μ*g /mL) activities.* In vivo*, the results proved that ethyl acetate extract at doses of 400 mg/kg bw decreased significantly the blood glucose level and lipid profile levels and increased the activities of antioxidant enzymes. These protective impacts of* Anthyllis henoniana* ethyl acetate flowers extract were confirmed by histological results.

**Conclusion:**

This study demonstrates, for the first time, that* Anthyllis henoniana* flowers ethyl acetate extract is effective in inhibiting hyperglycemia and oxidative stress caused by diabetes.

## 1. Introduction

Nowadays, diabetes mellitus (DM) has increased because of sedentary lifestyle and nutrition behavior changes in many countries, especially developing ones. This disease, comprising almost 90% of all cases of DM, is anticipated to rise regarding the number of diabetic people which is also expected to increase badly and reach above 80 million diabetes case by 2025 [1]. DM is a chronic metabolic irregularity caused by an imprecise balance of glucose homeostasis [2] that may cause death to humans. The digestive mechanism combines two steps, it starts by using the *α*-amylase enzyme that hydrolyzes the polysaccharides to oligosaccharides such as sucrose and then the intervention of the other enzymes (such as *α*-glucosidase, maltase, and sucrase) catalyzes the final step in carbohydrate hydrolysis to discharge the absorbable monosaccharides such as glucose. Previous researches have proved that *α*-glucosidase and *α*-amylase inhibitors are apt to DM [3]. The degradation of polysaccharides is carried on in a fast way and conducts to an ascent of the postprandial hyperglycemia levels. The adjustment of this activity has been demonstrated in the small intestine by human pancreatic *α*-amylase and it can be used as an aspect of DM therapy [4]. Otherwise, preceding studies have displayed that the oxidative stress is contributed as a result of persistent hyperglycemia, causing a decrease in the enzymes antioxidant activity and the excessive production of free radicals [5, 6] and the substances that are capable of reducing oxidative stress and postprandial hyperglycemia can be therapeutic for diabetes [7]. Furthermore, various phenolic compounds were able to inhibit* in vitro α*-glucosidase activity [8, 9] and to protect the cellular system against the oxidation damage [10], thus what leads a survey about the secondary metabolic content. The main issues behind drugs treatment like acarbose are the side effects such as flatulence, abdominal distention, meteorism, and possibly diarrhea [11]. This threat increases the demand for the use of natural substances containing antipostprandial hyperglycemia activity which certainly provide less or without side effects than the use of oral hypoglycemic and insulin. Previous studies supported and showed the link between the inhibitory potential of key carbohydrate-digesting enzymes, the oxidative stress, and the secondary metabolites contents from natural sources, such as the crude extracts of marine seaweed* Turbinaria ornata*, avocado pears leaves and fruit [12, 13], the hydromethanolic extract of leaves of* Emblica officinalis Gaertn* [14], and also numerous extracts of these plants (*Cinnamomum zeylanicum, Crataegus oxyacantha, Hibiscus sabdariffa, Morus alba, Portulaca oleracea, Rubus fruticosus, Syzygium aromaticum, Teucrium polium, Trigonella foenum-graecum, and Vaccinium arctostaphylos*) [15]. With the intention to identify new plant species in this antihyperglycemia category through specific inspection, the unexplored plant species* Anthyllis henoniana* (Coss.) has been investigated. Moreover, there are no specific scientific reports, nor specific references to* Anthyllis henoniana* constituents influence on the carbohydrate-digesting enzymes. It is a woody Saharan plant, located in the big desert of North Africa and belongs to the genus* Anthyllis* (Fabaceae). It is appropriate for the rehabilitation of deteriorating area [16] and one of the potential candidates interesting for protection against wind erosion and improvement of the pastoral value [17]. This research is focused on the valorization of this unstudied Saharan plant and the investigation of its phenolic compounds, potential antioxidant, antidiabetic activity, and its protective effects against liver and kidney toxicities in diabetic rats.

## 2. Materials and Methods

### 2.1. Collection and Extraction of Plant Material

*Anthyllis henoniana* flowers were collected from the Sahara of Beni Khedache Medenine Tunisia, approximate to this GPS coordination 33°13′09.6′′N 10°12′46.2′′E. The botanical identification was done in the botany laboratory of the Faculty of Sciences, University of Sfax, Tunisia, by Dr. Zouhaier NOUMI. A voucher specimen (N° LCSN 140) was created and deposited at the Laboratory of Organic Chemistry, Chemistry of Natural Substances herbarium, Department of Chemistry, Faculty of Sciences of Sfax.

100 g of* Anthyllis henoniana* flowers was dried, milled into a thin powder, and got extracted sequentially in the order with hexane, ethyl acetate, and methanol. All extracts were filtered and evaporated using a rotavapor at 40°C to identify extraction yields. Thereafter, the three collected extracts: hexane extract (HEx), ethyl acetate extract (EAEx), and methanol extract (MEx) were kept at low temperature until analysis.

### 2.2. Preliminary Phytochemical Screening

Phytochemical screening was done to identify the presence of secondary metabolites in according to the Harborne methods [18].

### 2.3. *In Vitro* Study

#### 2.3.1. Total Phenol, Total Flavonoid, and Tannin Contents

The total phenol content of* Anthyllis henoniana* flowers extracts was resolved using the method of Folin-Ciocalteu presented by Singleton and Rossi [19]. 0.5 mL of Folin-Ciocalteu reagent and 5 mL of Na_2_CO_3_ (20%) were added to 0.1 mL of each extract. After 30 minutes, the absorbance of each mixture was measured at 727 nm. Gallic acid was used as a standard. The phenol content in the extracts was expressed in terms of gallic acid equivalent, GAE (mg of GA/g of extract). The three extracts were analyzed in triplicate. The total flavonoid content of* Anthyllis henoniana* flowers extracts was identified by the aluminum chloride method reported by Zhishen et al. [20]. 0.3 mL of NaNO_2_ (5 %) and 4 mL of water were added to 1 mL of each extract. The mixture allowed standing for 5 min, followed by the addition of 0.3 mL of AlCl_3_ (10 %) then 2 mL of NaOH (1 M). The mixture was adjusted to 10 mL with water. Absorbance was measured at 510 nm and Quercetin was used as a standard. The three extracts were analyzed in triplicate.

Condensed tannin content was assessed using the vanillin assay [21], and 50 *μ*L of each extract was added to 1.5 mL of vanillin (4 %) and 750 *μ*L of HCl. After 20 min at room temperature, the absorbance was calculated at 500 nm. Results were expressed as milligram Catechin equivalents per gram of extract. The three extracts were analyzed in triplicate.

#### 2.3.2. Antioxidant Capacity Estimation

*(1) DPPH Inhibition Assay. *This assay was achieved by using the method of Ak and Gülcin [22] with slight modification. 1 mL of each concentration of test substances in ethanol (625-1250-2500–5000 *μ*g/mL) was added to 2 mL of the 0.004% (v/v) solution of DPPH in ethanol by dissolving 4 mg of DPPH in 100 mL ethanol and the mixture was stirred vigorously. After 30 minutes of incubating the tubes in dark at room temperature, the absorbance was taken at 517 nm. The data were compared with the controls, which contained 1 mL of 95% ethanol instead of the extract and vitamin E was used as positive control. The inhibition percentage was measured by

(1) $$\begin{array}{c} Inhibition\;\;\%=\left[\;1-\left(\;\frac{A\;\;sample}{A\;\;control}\;\right)\;\right]\times 100 \end{array}$$

*(2) Reducing Power Assay (FRAP). *The reducing power of* Anthyllis henoniana* flowers extracts was identified using the Oyaizu et al.'s method [23]. Briefly, extracts (1mg) were dissolved in 1 mL of distilled water and added to a mixture of 2.5 mL of potassium ferricyanide [K_3_Fe(CN_6_)] (1% w/v) and 2.5 mL phosphate buffer (0.2 M). After 20 min of incubation at 50°C, 2.5 mL of a (10% w/v) trichloroacetic acid was added to the mixtures, followed with centrifugation the samples at 3000 rpm for 10 min. Finally, 2.5 mL of the supernatant solution was merged with 0.5 mL of the 0.1% (w/v) solution of ferric chloride (FeCl_3_) and 2.5 mL of distilled water. The absorbances were analyzed spectrophotometrically at 700 nm, and vitamin E was the chosen standard. Otherwise, the detected increase in absorbance of the three samples demonstrated the reducing power elevation.

#### 2.3.3. Total Antioxidant Capacity (TAC)

The evaluation of total antioxidant capacity was assessed by the Prieto et al.'s method of [24]. The absorbance was determined at 695 nm against a blank after cooling the reaction at room temperature. Total antioxidant capacity was expressed as equivalents of vitamin E which was used as a standard.

#### 2.3.4. Determination of *α*-Amylase Activity* In Vitro* by a CNPG3 Method

The *α*-amylase inhibition analysis was analyzed according to the method mentioned by Hui et al. [25]. 400 *μ*L of 0.1M sodium phosphate buffer and 500 *μ*L of starch solution 1 % were added to 50 *μ*L of sample solution or standard (acarbose) and 50 *μ*L of enzyme solution (4 U/mL). The incubation of this mixture was carried out at 37°C for few minutes and then 3 mL of 3.5-dinitro salicylic acid was included as a reagent. This mixture was heated to boiling for 10 min before the addition of 20 mL of distilled water. Absorbance measurement was done at 540 nm and the inhibition percentage was calculated utilizing this formula:

(2) $$\begin{array}{c} Inhibition\;\%=\left[\;1-\left(\;\frac{A\;\;sample}{A\;\;control}\;\right)\;\right]\times 100 \end{array}$$

IC_50_ values were resolved from the scheme of inhibition percentage against sample concentration and acarbose was used as a specific standard. All tests were made in triplicate.

#### 2.3.5. The Determination* In Vitro* of *α*-Glucosidase Activity

*(1) Isolation. *According to Nishioka et al. [26], the crude mice intestinal extract was prepared with minor modifications. After the dissection of 6 mice, small intestines were taken and then were washed with a normal saline solution (sodium chloride 0.9 %) and then with 0.1M potassium phosphate buffer with 5 mM EDTA. The mucosa was scraped from these small intestines and was mixed with potassium phosphate buffer before centrifugation at 21.000g for 1h. The precipitate was stirred during 30 min, after the complete dissolving, in 0.1 M potassium phosphate buffer and 1% TritonX-100 solution. The mixture has been centrifuged at 20.000 g for 60 min and the supernatant was dialyzed in 0.01M potassium phosphate buffer for 24 h.

*(2) α-Glucosidase Inhibitory Activity Assay. *According to Ranilla et al. [27], the enzyme activity was carried on using PNP-glycoside as a substrate in 0.1M potassium phosphate buffer. This activity was identified by calculating p-nitrophenol released from PNP-glycoside at 400 nm. All reactions were performed at 37°C for a period of 30 min with three replicates.

(3) $$\begin{array}{c} Inhibition\;\;\%=\left[\;1-\left(\;\frac{A\;\;sample}{A\;\;control}\;\right)\;\right]\times 100 \end{array}$$

IC_50_ values were resolved from the scheme of inhibition percentage against sample concentration and acarbose was used as a specific standard. All tests were made in triplicate.

### 2.4. *In Vivo* Study

#### 2.4.1. Experimental Animals

The analysis of the current study was carried out with 32 adult healthy male Wistar rats, weighing 180±20 g. Rats were received from animal house of the local Central Pharmacy, Tunisia. The animals were retained and conditioned in a breeding chamber with the natural controlled system (room temperature and a natural 12 h-12 h light-dark cycle). A standard diet and a tap water were furnished.

#### 2.4.2. Acute Toxicity Test

The procedure of acute oral toxicity test was carried out using the classic procedure of the acute toxic. The animals were branched into six groups of seven rats each and were given the ethyl acetate flowers extract of* Anthyllis henoniana *at the doses of 100, 200, 500, 3000, 1000 mg/kg, and 2000 orally in a volume of 10 mL/kg and the animals. All rats were maintained under regular observation for mortality and behavioral changes during 24 h at 72 h [28].

#### 2.4.3. Induction of Diabetes

Diabetes was treated by an intraperitoneal injection of alloxan (150 mg/Kg bw) dissolved in normal saline. The alloxan injection may produce a fatal hypoglycemia as a result of a high reactive release of pancreatic insulin; that is why the rats were also orally given 5–10 mL of a 20% glucose solution after six hours. These rats were then kept with free access to a 5% glucose solution for one day to avert the intense hypoglycemia. The development of diabetes has been checked by measuring serum glucose level after four days of alloxan injection. DM was confirmed by elevated fasting serum glucose over 300 mg/dL [29].

#### 2.4.4. Experimental Design

The experimental animals were branched into 4 groups (n=8) as follows:

Group 1: normal control rats (cont) which were given a balanced diet (Cont).

Group 2: untreated diabetic control rats (Diab).

Group 3: diabetic rats treated with the ethyl acetate flowers extract of* Anthyllis henoniana* extract at a dose (400 mg/kg bw/day) in 1 mL by oral gavage for 28 days (Diab+EAEx).

Group 4: diabetic rats provided and treated with the standard diabetic drug (acarbose, 12 mg/Kg bw/day) in 1 mL by oral gavage for 28 days (Diab+Acar).

After 28 days of treatment, all rats from each experimental group were weighted and after an overnight fast. Their trunk blood was collected from the spots of decapitation for the serum preparation. The kidney, liver, and pancreas were detached and managed to be fat-free. Pieces from these organs were maintained in a Bouin solution for one day and then planted in paraffin. 5-*μ*m sections were taken to perform hematoxylin-eosin staining. The slides were photographed optically with an Olympus CX41 microscope equipped with a camera.

#### 2.4.5. Time Course of Changes in Blood Glucose Levels

Blood glucose concentrations of 12h fasted rats groups were measured at 8:00 am before starting the feeding program with glucose oxidase-peroxidase method. The fasting blood glucose levels were determined on 1, 7, 14, 21, and 28 days, regularly.

#### 2.4.6. Measurements of Body Weights

All measurements of the body weight were estimated in 1, 7, 14, 21, and 28 days, regularly. Rats were weekly weighed individually using a precision balance to measure the weekly body weight gains.

#### 2.4.7. Oral Glucose Tolerance Test (OGTT)

According to Kolsi et al. [30], this test demonstrates the capacity to reply suitably to a glucose challenge. Oral glucose tolerance test was performed in control and treated rats groups, 24 h before their decapitation. A glucose load (2 g/kg bw) was given to each rat from all consisting groups by gastric gavage path. Blood samples were assembled from the tail vein at 0, 30, 60, 120, and 240 min regularly and their glucose levels were automatically measured using a one-touch glucometer.

#### 2.4.8. Analysis of Lipid Profile

The concentrations of serum lipids level of triglycerides TG, total-cholesterol T-Ch, low-density lipoprotein-cholesterol LDL-c, and high-density lipoprotein-cholesterol HDL-c were measured using the standard commercial kits (Biolabo) on an automatic biochemistry analyzer (BS-300) at the biochemical laboratory of Hospital of Hedi Chaker of Sfax. Low-density lipoprotein-cholesterol concentration was identified basing on Friedewald et al. formula [31].

#### 2.4.9. Biochemical Analysis

The analysis of serum lipase pancreatic, *α*-amylase, alanine aminotransferase (ALT), aspartate aminotransferase (AST), creatinine, and urea rates were measured using commercial kits from Biolabo (France). The manufacturer's protocols and instructions have been followed to perform the current assays.

#### 2.4.10. Antioxidant Activity

All different used organs tissues (Kidney, pancreas, and liver) were measured, grinded, and homogenized in 5 mL of phosphate buffer and centrifuged at 3500 rpm for 30 min at 4°C. After the collection of the supernatant, the amount of thiobarbituric acid reactive substances (TBARS) was measured according to Yoshioka et al. method [32] and expressed as nmol MDA/mg protein and AOPP oxidation of protein levels was identified using Kayali et al. method [33] and expressed as nmol/mg protein.

The activity of superoxide dismutase (SOD) was examined by Marklund and Marklund method [34] and expressed as U/mg protein. Reduced glutathione (GSH) activity was estimated according to the described method by Paglia and Valentine [35] and expressed as ug GSH/mg protein. The catalase (CAT) activity was analyzed colorimetrically at 240 nm and expressed as mmoles of H_2_O_2_ consumed/min/mg protein as described by Aebi [36]. The determination of total protein level was processed through the procedure of Lowry et al. [37] using BSA (bovine serum albumin) as the standard at 660 nm.

#### 2.4.11. Histopathological Study

After removing liver; pancreas; and kidney, small pieces of these organs were fixed in Bouin solution for one day. After an aqueous wash, the tissues were dehydrated using an ascendant gradient of ethanol and then were cleared utilizing xylene or toluene. Finally, the tissues were embedded in paraffin before being sliced into sections of 3-5 mm thickness and stained with hematoxylin and eosin.

### 2.5. Statistical Analysis

Results are presented as mean ±SD from three replicates of each experiment. Statistical analysis was performed using one-way analysis of variance (SPSS software) and the significant differences between means were determined by Student's t-test. The values of p < 0.05 were considered significantly different. Pearson correlation coefficient was calculated to establish the relationship between antioxidant activity and polyphenols, flavonoids, and condensed tannins contents.

## 3. Results

### 3.1. Phytochemical Screening

Phytochemical screening of all* Anthyllis henoniana* flowers extracts proved the absence of alkaloids, quinones, and tropolones (Table 1). However, both of methanol and ethyl acetate extracts tested positive for the existence of flavonoids and tannins. Sterols and terpenoids marked their presence in hexane and ethyl acetate extracts.

### 3.2. Total Phenol, Flavonoid, and Tannin Contents

**Figure 1:**
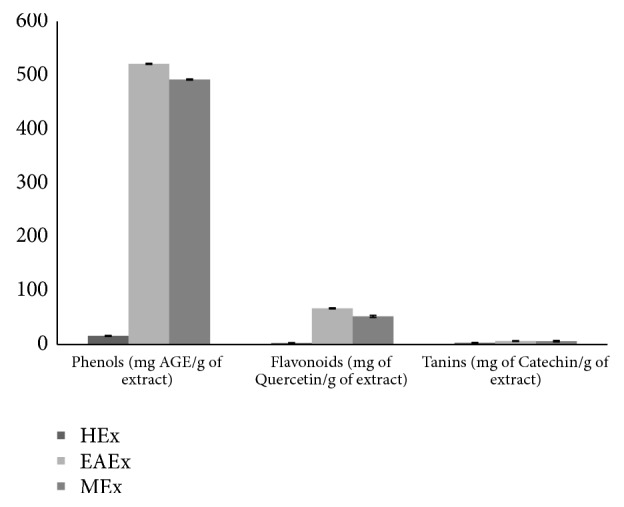
Total phenols, flavonoids, and tannins contents of flowers extracts of* Anthyllis henoniana*. All the values are means ± SD (n=3). HEx: hexane extract; EAEx: ethyl acetate extract; MEx: methanol extract.

The main secondary metabolites, including phenols, flavonoids, and tannins contents in the three extracts (ethyl acetate, methanol, and hexane), are shown in Figure 1. Phenols content was measured using Folin-Ciocalteu reagent in terms of gallic acid equivalent (standard curve equation: y = 9.221x + 0.2, r^2^= 0.955). The ethyl acetate extract possessed the highest content of total phenols recorded of value 521.08 ± 0.94 mg of GAE/g as compared to methanol extract of value 491.9 ± 0.96 mg of GAE/g. However, hexane extract was recorded as 16.06 ± 0.76 mg of GAE/g. Total flavonoid amount was explicitly referred in terms of Quercetin equivalent (the standard curve equation: y = 12.57x + 0.106, r^2^ = 0.996); the ethyl acetate extract exhibited the maximum of flavonoid content and corresponded to 67.20 ± 0.97 mg of Quercetin/g, followed by methanol and hexane extracts as showed, respectively, 52.31 ± 1.85 mg of Quercetin/g and 2.89 ± 0.68 mg of Quercetin/g. The condensed tannins contents of the three extracts were expressed in terms of Catechin equivalent (the standard curve equation: y = 7.026x – 0.0191, r^2^ = 0.999).* Anthyllis henoniana* flowers extracts contained a low quantity of the condensed tannins present, respectively, in the ethyl acetate extract (6.57 ± 0.56 mg of Catechin/g), methanol extract (6.29 ± 1.29 mg of Catechin/g), and hexane extract (3.18 ± 0.55 mg of Catechin/g).

### 3.3. Antioxidant Activity by DPPH Assay

**Figure 2:**
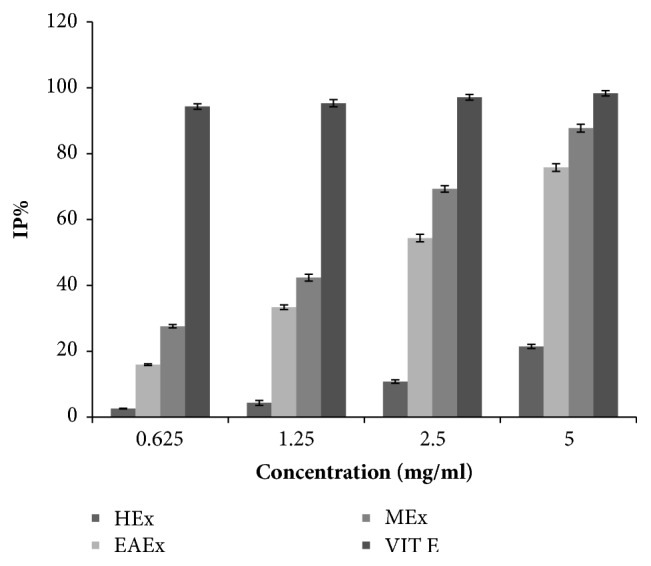
Antioxidant activity by DPPH method of flowers extracts of* Anthyllis henoniana *at different concentrations. All the values are means ± SD (n=3). HEx: hexane extract; EAEx: ethyl acetate extract; MEx: methanol extract.

As evident from the Figure 2, the radical scavenging activity of the extracts and the standard increased with the concentration elevation; at the maximum concentration (5000 *μ*g/mL), all the extracts gave lower inhibition percentages than the vitamin E (98.32 ± 0.82%). The methanol extract had the highest DPPH radical inhibition activity (87.74 ± 1.19%) followed by the ethyl acetate extract (75.8 ± 1.15%) and hexane extract (21.48 ± 0.63%). Otherwise, the comparison of IC_50_ (mg/mL), which is the necessary amount of each sample to decline by 50% the absorbance of DPPH, is already presented in Table 2 in this decreasing order: vitamin E (IC_50_ = 0.2 mg/mL) > methanol extract (IC_50_ = 1.58 mg/mL) > ethyl acetate extract (IC_50_ = 2.34 mg/mL). However, the hexane extract with a maximum inhibition percentage at a maximum concentration did not reach 50% of DPPH radical inhibition; this result may be due to the low antioxidant activity of this extract.

### 3.4. Ferric-Reducing Antioxidant Power Assay (FRAP)

**Figure 3:**
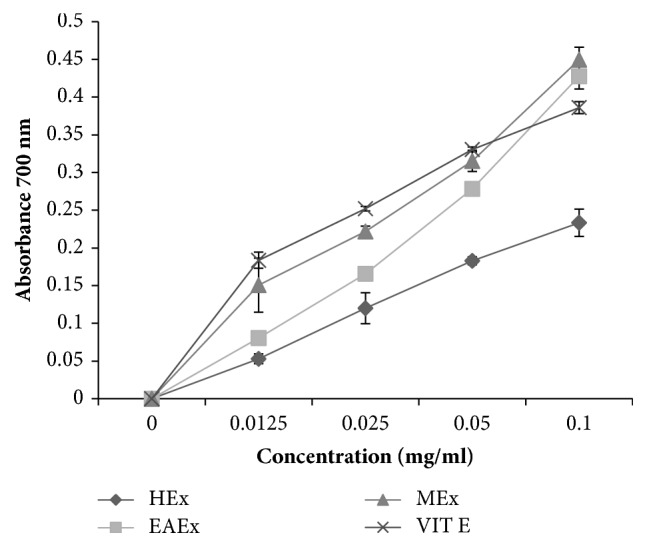
Reducing power of flowers extracts of* Anthyllis henoniana*. All the values are means ± SD (n=3). HEx: hexane extract; EAEx: ethyl acetate extract; MEx: methanol extract.

FRAP is used to assess the ability of an antioxidant to give an electron and prevent from peroxidation of linoleic acid, which is due to the existence of reductones.  Figure 3 shows the reducing powers of the three extracts. At 1 mg/mL concentration, the methanol extract reduced the (Fe^3+^) to ferrous ions (Fe^2+^) more effectively (absorbance = 0.449±0.016) than ethyl acetate extract (absorbance = 0.427±0.017) and hexane extract (absorbance = 0.233±0.018). The reducing power of the extracts and the standard increased with increasing concentration. Among all the three extracts, only the hexane extract has shown the lowest reducing power. Besides, the ethyl acetate extract and methanol extract have demonstrated higher maximum absorbance than vitamin E which is used as a reference.

### 3.5. Total Antioxidant Capacity TAC

TAC was measured by a spectrophotometric method using a phosphomolybdenum method, which was based on the reduction of Mo^6+^ to Mo^5+^ by the sample analytes and the subsequent formation of green phosphate/ Mo^5+^ compound with a maximum absorption at 695 nm. Sharp absorbance values proved the possession of significant antioxidant activity. TAC was identified using the standard curve of vitamin E (equation: y = 2.046x + 0.043, r^2^= 0.991) and revealed that the methanol extract (59.33 mg vitamin E/g extract) is endowed with the highest antioxidant capacity followed by ethyl acetate extract (50.61 mg vitamin E/g extract) and hexane extract (6.13 mg vitamin E/g extract), respectively (Table 2).

### 3.6. Correlation between Phenolics Contents and Antioxidant Activity

To prove the contribution and the role of phenolic, flavonoid, and tannins contents in* Anthyllis henoniana* flowers extracts on antioxidant activity, the Pearson correlation coefficient (*r*^2^) was demonstrated in Table 3. The results showed the presence of a significant linear correlation between antioxidant activities determined by using DPPH and TAC methods and phenols, flavonoids, and tannins contents, respectively. The strongest correlative value is obtained with DDPH and phenols contents (*r*^2^ = 0.72). These results presented a good correlation between phenolic compounds with antioxidant activities of* Anthyllis henoniana* flowers extracts.

### 3.7. *In Vitro* Antipostprandial Hyperglycemia Studies

#### 3.7.1. *α*-Amylase and *α*-Glucosidase Inhibitory Potentials

**Figure 4:**
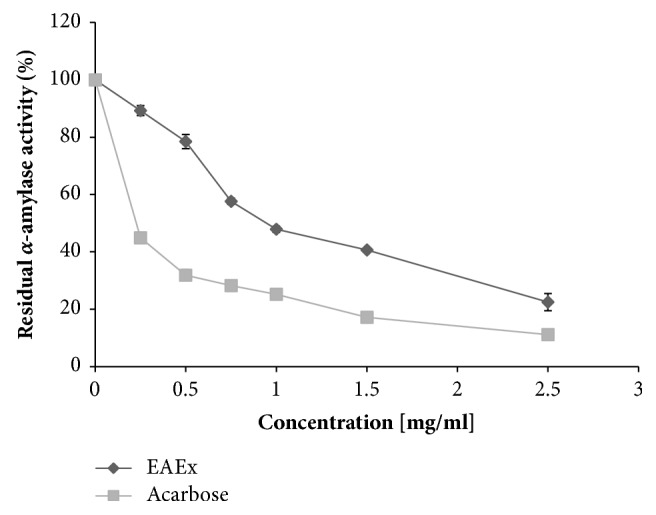
*α*-amylase activity (%) of the ethyl acetate flowers extract (EAEx) of* Anthyllis henoniana* at different concentrations compared with acarbose. All the values are means ± SD (n=3)

**Figure 5:**
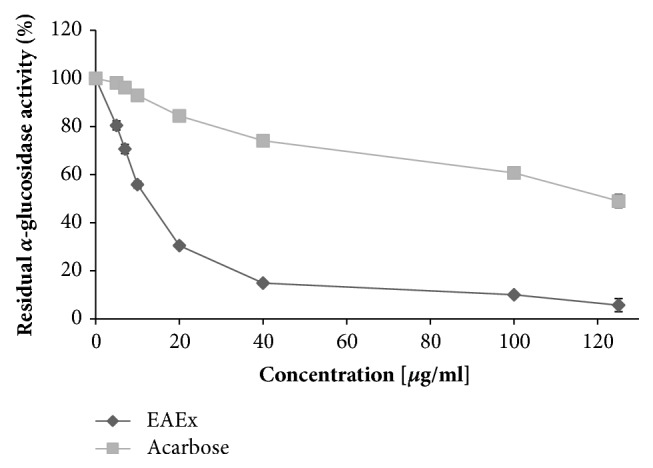
*α*-glucosidase activity (%) of the ethyl acetate flower extract (EAEx) of* Anthyllis henoniana *at different concentrations compared with acarbose. All the values are means ± SD (n=3).

The results demonstrated that the screening of all three flowers extracts of* Anthyllis henoniana* had been performed, in order to identify which one(s) have an antiamylase activity by using pancreatic amylase as a model. Antiamylase activity was expressed as inhibition percentage of three extracts, the hexane extract has the lowest result (7%), then the methanol extract (18.4%), and the highest result was established for the ethyl acetate extract by 72.45%. Thus, the focus was directed to the EAEx. The inhibition analysis of *α*-amylase and *α*-glucosidase was performed using acarbose as a positive control. The *α*-glucosidase inhibition assay presented that the enzyme was potently inhibited by ethyl acetate extract with the IC_50_ value of 17 *μ*g/mL, in resemblance with acarbose (125 *μ*g/mL) (p<0.001). Interestingly, in the *α*-amylase inhibition assay, the inclusion of the* Anthyllis henoniana* EAEx with different concentrations reduced successfully the residual *α*-amylase activity which was less effective in inhibiting *α*-glucosidase than acarbose drug. EAEx showed an IC_50_ value of 920 *μ*g/mL in resemblance with acarbose (IC_50_ of 220 *μ*g/mL) (p<0.001). The important* in vitro* inhibitory potential of ethyl acetate extract compared to the other two extracts guides to its use for further* in vivo* study (Figures 4 and 5).

### 3.8. Acute Toxicity Studies

The toxicity studies demonstrated that ethyl acetate extract from* Anthyllis henoniana*'s flowers does not mark any sign of toxic reactions or mortality up to a dose of 2000 mg/kg bw. The observation of rats behavior was made at an interval of every 8 h during 72 h.

### 3.9. Effect of* Anthyllis henoniana* Flowers Extracts on Body Weight and Blood Glucose Levels

**Figure 6:**
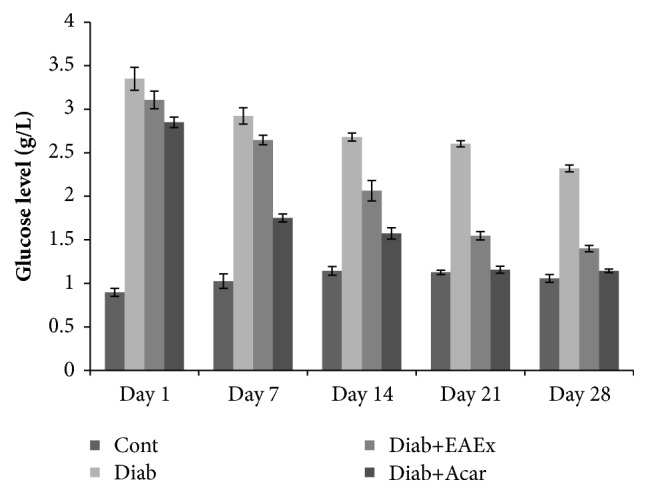
Blood glucose levels of different experimental groups of rats. Control group (Cont) and diabetic control (Diab). Diab+ EAEx: diabetic rats treated with EAEx of* Anthyllis henoniana *at 400 mg/kg bw per day for 28 days; Diab+Acar: diabetic rats treated with acarbose at dose of 12 mg/kg bw per day for 28 days. The values are means ±SD (n=8 for each group).

As shown in Table 4 and Figure 6, diabetic rats presented significant decreases (p<0.001) in body weights by -31.5% as compared to control rats. However, treated diabetic rats by ethyl acetate extract (at dose 400 mg/kg bw) and with acarbose displayed significant increases in body weights by + 35.28% and +38.50%, respectively. Moreover, we had noticed significant increases (p< 0.001) in blood glucose in diabetic rats by +54.47 % compared with the control groups. Otherwise, the administration of ethyl acetate extract at doses of 400 mg/kg bw demonstrated a significantly (p<0,001) decrease in blood glucose level by -40.08% as compared to diabetic rats.

### 3.10. Effect of* Anthyllis henoniana* Flowers Extraction OGTT in Diabetic Rats

OGTT has been performed in responsive fasted rats after oral administration of ethyl acetate extract to prove its outcome and affirm the potential inhibitory effect of digestive enzymes on carbohydrates digestion.

The results clearly showed that acute oral administration of EAEx (400 mg/kg bw) presented an important decrease of glucose concentration peak 60 min after glucose administration, as compared to diabetic rats (Table 5).

### 3.11. Effect of Anthyllis henoniana Flowers Extract on *α*-Amylase and Lipase Activity in Serum

**Figure 7:**
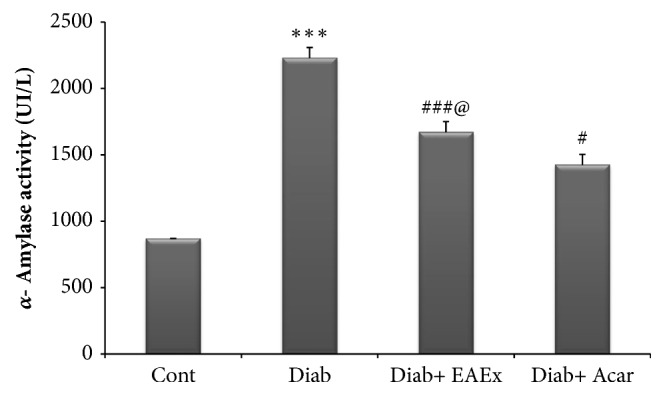
*α*-amylase activity in serum of different experimental groups of rats. Values represent mean ± SD (n = 8 for each group). Control groups (Cont) and diabetic control (Diab). Diab+ EAEx: diabetic rats treated with EAEx of* Anthyllis henoniana *at 400 mg/kg bw per day for 28 days; Diab+Acar: diabetic rats treated with acarbose at dose of 12 mg/kg bw per day for 28 days. Values differ significantly at ^*∗∗∗*^p< 0.001, as considered significant compared to control groups; ^#^p<0.05, ^###^p < 0.001, as considered significant compared with diabetic rats;^@^p <0.05, as considered significant compared with diabetic rats treated with acarbose.

**Figure 8:**
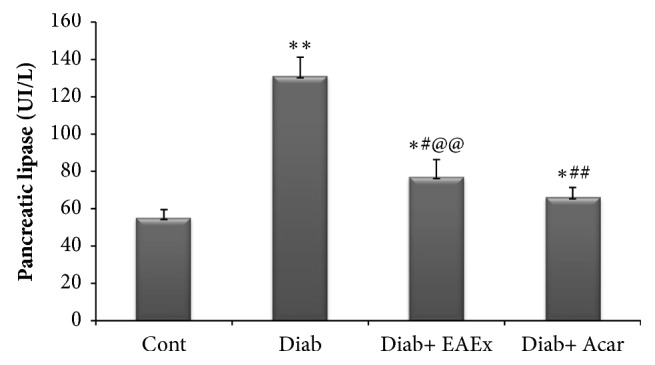
Lipase pancreatic level in serum of different experimental groups of rats. Values represent means ± SD (n = 8 for each group). Control groups (Cont) and diabetic control (Diab). Diab+ EAEx: diabetic rats treated with EAEx of* Anthyllis henoniana *at 400 mg/kg bw per day for 28 days; Diab+Acar: diabetic rats treated with acarbose at dose of 12 mg/kg bw per day for 28 days. Values differ significantly at ^*∗*^p <0.05,^*∗∗∗*^p < 0.001, as considered significant compared to control groups;^#^p<0.05^##^_,_ p<0.01, and ^###^p < 0.001, as considered significant compared with diabetic rats; ^@^p<0.05, ^@@^p<0.01, and ^@@@^p < 0.001, as considered significant compared with diabetic rats treated with acarbose.

As comparing the diabetic rats with a control group in both Figures 7 and 8, the activities of both *α*-amylase and lipase pancreatic in the serum of diabetic rats presented significant elevation by +57.03% and +40.71% accordingly. However, the ethyl acetate flowers extract of* Anthyllis henoniana* administration to diabetic rats presented a remarkable contraction in serum *α*-amylase and lipase activities by -27.70% and by -38.33%, respectively.

### 3.12. Effect of Anthyllis henoniana Flowers Extracts in Lipid Profile

This study showed an important rise in lipid profile parameters (T-CH, TG, LDL-c, and HDL-c) in diabetic rats as compared to control group (Table 6). The treatment with ethyl acetate flowers extract of* Anthyllis henoniana* (400 mg/kg bw) revealed significant decreases in T-CH by -12.43%, TG by -71.27%, and LDL-c by -39.39% and an increase in HDL-c by +152% as compared to diabetic rats.

### 3.13. Effect of Anthyllis henoniana Flowers Extract in Hepatic Dysfunction

The results showed increases in markers of oxidative stress as TBARS and AOPP by + 64.19 % and +76.11% in diabetic rats as compared to control groups (Table 8). The indices of hepatic dysfunction parameters ALT and AST activities obtained in serum of diabetic rats were also increased by +34.58%, +40.63, respectively (Table 7). The results presented a fascinating decrease in the CAT, SOD, and GSH activities in hepatic tissues of diabetic animals compared to controls groups (Table 8). Diabetic rats given ethyl acetate flower extract of* Anthyllis henoniana* at doses of 400 mg/kg bw showed an apparent protective effect in hepatic function by decreasing significantly (p<0,001) in AST and ALT by -29.50% and -47.68% as compared with diabetic rats and aimed to adjust the antioxidant effectiveness of hepatic function also to fix the transformed values by reaching the normal levels again.

**Figure 9:**
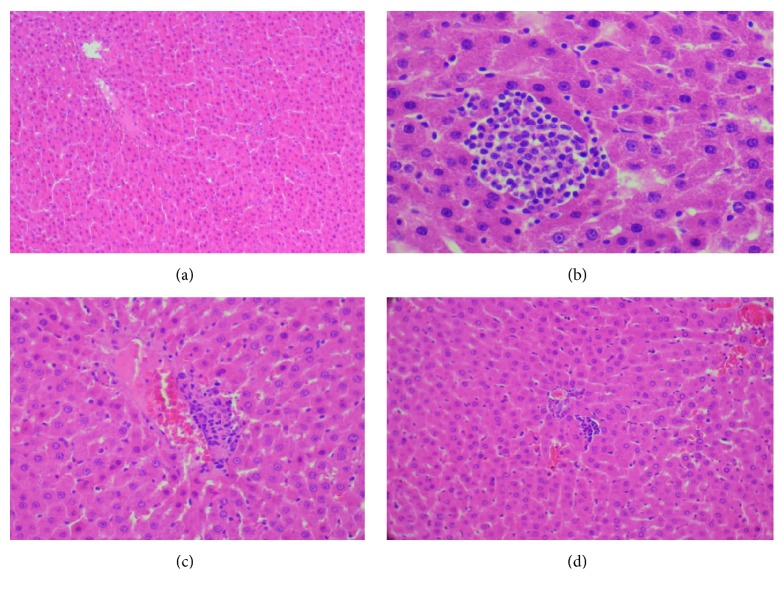
Histopathological observation of liver in the control and experimental groups of rats. Section of the liver from a control rat, (a) showing normal architecture, (b) liver of diabetic rat showing a moderate vacuolation of hepatocytes and infiltration of lymphocyte cells, (c) liver of diabetic rat treated with EAEx (400 mg/kg bw) of* Anthyllis henoniana *showed a potential protective effect, and (d) diabetic rat treated with acarbose protective action was shown (H&E ×400).

All of these findings were associated with the histological sections of the liver which showed a tolerable vacuolation of hepatocytes in diabetic rats (Figure 9(b)) while the treatment with ethyl acetate extract (400 mg/kg bw) and acarbose showed a protective effect (Figures 9(c) and 9(d)).

### 3.14. Effect of Anthyllis henoniana Flowers Extract in Pancreas Dysfunction

In diabetic rats, TBARS and AOPP levels were elevated by +64.91% and +43.44%, respectively. However the antioxidant enzymes activities such as CAT, SOD, and GSH had endured important decreases by -10.36%, -146.61%, and -231.84%, as compared with the control group (Table 8).

**Figure 10:**
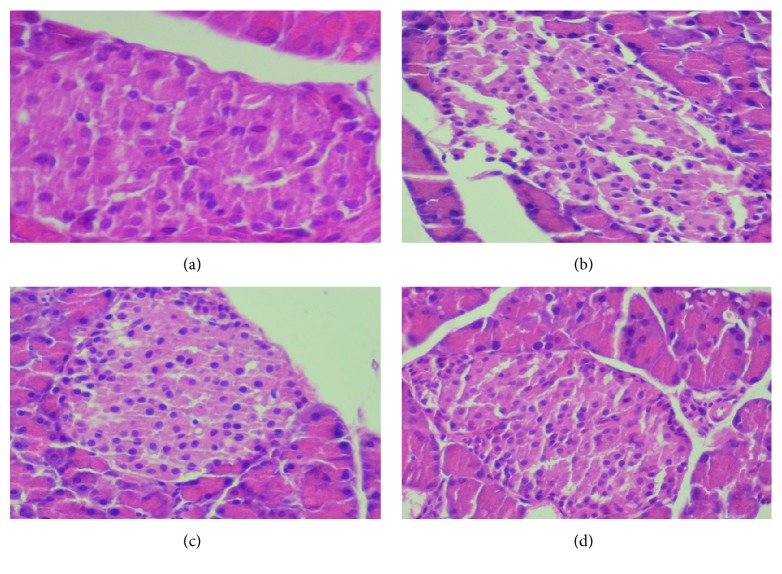
Histopathological observation of pancreatic islet cells in the control and experimental groups. (a) Control group of rat, the pancreas shows normal pancreatic islet cells, (b) diabetic rats pancreas, showing a distorted and atrophic islet of Langerhans displaying cells with vacuolated cytoplasm, (c) in treated diabetic rats with EAEx of* Anthyllis henoniana *(400 mg/kg bw), showing normal islet of Langerhans with numerous *β* cells, and (d) in diabetic rats treated with acarbose, showing a distorted islet of Langerhans displaying cells with vacuolated cytoplasm (H&E ×400).

By oral administration of the ethyl acetate extract (400 mg/kg bw) and acarbose, the antioxidant ability of pancreas was regulated and the concerned values reached the normal levels. The histological study confirmed these results and disclosed the protective action of the pancreas *β*-cells of diabetic rats given by the administration of the ethyl acetate extract (400 mg/kg bw) (Figure 10(c)).

### 3.15. Effect of Anthyllis henoniana Flowers Extract in Renal Dysfunction

**Figure 11:**
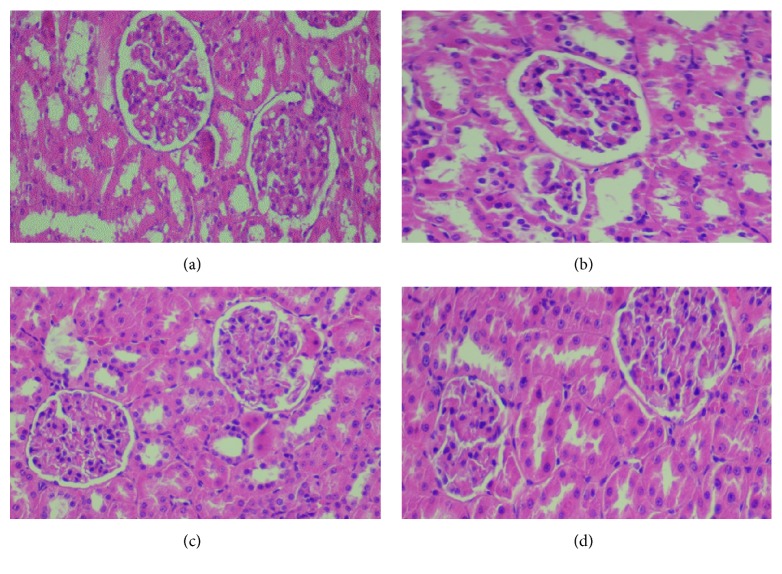
Histopathological observation of kidney in the control and experimental groups of rats. (a) Section of the kidney from a control rat, (b) diabetic rat showing Bowman's space size, (c) diabetic rat treated with EAEx (400 mg/kg bw) of* Anthyllis henoniana* showed a potential protective effect, and (d) diabetic rat treated with acarbose demonstrated also a potential protective effect was shown (H&E ×400).

In diabetic rats, the SOD, CAT, and GSH activities in the kidney decreased by -228.45 %, -10.36%, and-136.11%, respectively as compared to control group. Moreover, an important increase in TBARS and AOPP in kidney and also renal dysfunction parameters observed in creatinine and urea level in plasma by +34.34% and +45.93%, respectively, were acquired in alloxan-treated rats (Tables 7 and 8). The renal SOD, CAT, and GSH antioxidant activities increased after administration of ethyl acetate extract (400 mg/kg bw) as well as acarbose and showed also a decrease in TBARS and AOPP amounts (Table 8) and renal dysfunction indices levels in plasma. Observation of the histological sections of the kidneys of all experimental rats showed that the kidneys of the diabetic rats presented a structural alteration of the cortical zone affecting the Bowman capsules. These affected capsules showed an increase in Bowman's capsule space (Figure 11(b)). Kidney protection was remarkable due to the treatment with the ethyl acetate extract (400 mg/kg bw) and the drug acarbose which preserved the structure of the cortex and corrected the nephropathic damage compared to diabetic rats (Figures 11(c) and 11(d)).

## 4. Discussion

DM is one of the most common metabolic diseases; it is known as an increase of the blood glucose level and impaired metabolism of proteins and lipids. Currently, diabetes is considered as one of the most critical issues in the world. Much research performed more efforts to seek new natural antioxidant molecules, which are considered to be relatively safer and with less or without side effects. Recently, the attention is focused on plants which contain high concentrations of phytochemical compounds because of their potential health-promoting effects [38]. This study has investigated, for the first time, the potential antidiabetic and antioxidant effects of* Anthyllis henoniana* flowers extracts in metabolic disorders in alloxan-induced diabetic rats.

As compared to others extracts the ethyl acetate flowers extract of* Anthyllis henoniana* possessed the maximum of phenols and flavonoids contents which can be attributed to its good solubility, low toxicity, medium polarity, and high extraction capacity. Likewise, ethyl acetate tends to extract a way better than ethers and hexane and is known to be more natural and safer than other chemicals because it occurs in nature by contributing to the characteristic aroma of several fruits [39]. The extraction of phytochemical constituents may depend on the polarity and the molecular weight of the chosen solvent; these factors present a major role in natural substances isolation. According to Naczk et al. [40], the solubility of phenolics is affected by the polarity of used solvents, thus it is very difficult to develop an extraction procedure suitable for extraction of all plant phenolics. Overall, the findings indicated that the ethyl acetate extract of* Anthyllis henoniana's* flowers was rich in flavonoid and phenolic contents, which could be the main contributor to their antioxidative properties as many studies affirmed that flavonoids and phenols offered the highest ability of scavenging activity in medicinal plants [41, 42].

In this study, the results clearly stated that the methanol and ethyl acetate extracts which contained the highest amounts of total phenolic content had the strongest radical scavenging activities and total antioxidant capacities. The positive correlation between the phenolic compounds and the antioxidant activity using DDPH and TAC confirmed the hypothesis of the effective role of these types of compounds. Moreover, both of ethyl acetate and methanol extracts showed high activities in the reducing power at a maximum concentration of 1mg/mL. Miceli et al. [43] have reported similar results by revealing a positive relationship between total phenols, flavonoids, and the antioxidant activities (DDPH and FRAP). These results could be allotted to the presence of phenolic compounds, which play an efficient role as a hydrogen donator, reducing agents and singlet oxygen quenchers [44, 45]. Many plants from the Fabaceae family were used in traditional, complementary and alternative medicine [46, 47]. So, this research aims to seek the antidiabetic potential by determination of *α*-glucosidase inhibitory and *α*-amylase assay. The inhibitory effect of ethyl acetate extract against *α*-amylase showed a good concentration-dependent activity. Actually, *α*-amylase and *α*-glucosidase inhibitory activities were marked to elevate in a dose-dependent approach, with the observation of the highest inhibitory effect in the ethyl acetate extract.

Therefore,* in vivo* studies had been proceeding with the ethyl acetate extract and confirmed the role of alloxan injection in inducing hyperglycemia by +54.74% as compared to control groups. However, it was noted that the EAEx administration at doses (400 mg/kg bw) to diabetic rats inhibited* in vivo* blood *α*-amylase by involving an important blood glucose decrease. In fact, the inhibitory potential of this extract was quite similar to that established by acarbose. The presence of phytochemical compounds such as flavonoids, phenols, and tannins with important contents in the ethyl acetate extract could be related to the antidiabetic effect. Moreover, many glycosides have been assumed as potential antidiabetic agents due to their significant inhibitory effect against *α*-amylase and their role in DM prevention by including them in the dietary strategy [48, 49]. Other studies have suggested that inhibiting activity of different flavonoids on glucose absorption is possibly due to the competitive inhibition of sodium-dependent glucose transporter 1 [48, 50]; e.g., especially, isoquercitrin has shown in previous studies its hypoglycemic effect [51–53]. The current findings showed the ability of the ethyl acetate extract to reduce blood glucose level, due to the improvement of insulin action to improve glycemic control in peripheral tissue and to the inhibitory effects of intestinal disaccharidases. This extract (EAEx) presented a protective effect in pancreatic *β*-cell function and/or decreased *β*-cell mass and lytic changes in the pancreas islet induced by alloxan, as have been seen in the histological examination. DM causes a disorder in lipid metabolism which led more investigations to survey and understands the pathogenesis [54]. The hyperglycemia accompanied by a dyslipidemic disturbance proven by a significant elevation of TG, T-Ch, and LDL-c and a decrease in HDL-c in serum pancreatic lipase. Many studies had mentioned the danger caused by LDL oxidation which contributes to the formation of atherosclerosis plaque lesions [55]. After the ethyl acetate extract administration to diabetic rats, the blood glucose level has been lowered and the lipid metabolism has enhanced which caused a remarkable reduction in serums TG, T-Ch, and LDL-c and an elevation in HDL-c levels as compared respectively with diabetic rats. Increasing HDL-C or HTR ratios has been quoted among the most substantial criteria of antiatherogenic agents. There has been much research reported on the association between the low incidence of cardiovascular diseases and higher levels of HDL-c [56, 57]. The hypolipidemic action of ethyl acetate extract might be the result of the carbohydrate retardation and lipid absorption caused by the presence of biologically active compounds in the flowers extract; these results were similar to the study of Daisy and Rajathi [58] that showed the hypoglycemic and hypolipidemic effects of aqueous extract from flowers and leaves of* Clitoria ternatea Linn*. (Fabaceae) at doses of 400 mg/kg bw by decreasing in serum level of T-Ch, LDL-c and serum glucose, glycosylated hemoglobin, and insulin in diabetic rats. Besides the antidiabetic and antihyperlipidemic actions, the ethyl acetate extract administration prevents liver-kidney dysfunctions. AST and ALT, along with other blood plasma enzymes, were used to evaluate the hepatic dysfunction [59, 60]. The increased liver enzyme activities are biomarkers for hepatic disorder and the high increase of transaminases causes inflammation or hepatocellular disorders [61, 62]. These findings were in accordance with the current study, which was demonstrated by the increased hepatic levels in diabetic rats as compared to control groups. Otherwise, the ethyl acetate extract administration (400 mg/kg/bw) marked significant decreases in AST and ALT as compared to diabetic rats. The hepatoprotective effect of ethyl acetate extract was proved through histological results in liver tissues, by reducing the liver fat levels. Thus, the antioxidant compounds of the ethyl acetate flowers extract of* Anthyllis henoniana* decreased the oxidative stress and protected the hepatic. The result exhibited that the treatment with ethyl acetate extract (400 mg/kg bw) displayed an important increase in antioxidants enzymatic and nonenzymatic status (SOD, CAT, and GSH levels) and a decrease in TBARS and AOPP, respectively, as compared to diabetic rats. Besides the amelioration effect of ethyl acetate extract EAEx in liver toxicity, the administration of this extract and acarbose ameliorates the renal toxicity by decreasing the serum creatinine and serum urea rates as compared to diabetic animals.

Furthermore, the antioxidant activity performed a remarkable increase in SOD, CAT, and GSH activities in the kidney of treated diabetic rats with EAEx (400 mg/kg bw), respectively. The positive effect of ethyl acetate extract was proved by histological reporting in kidney tissues by reducing the increase of glomerular condensation and the capsular space. The antihyperglycemic activity of this extract has been associated with its flavonoids and total phenolic contents which may contribute to protective property of ethyl acetate extract for the complication of the liver and kidney toxicity against alloxan-induced diabetic rats.

## 5. Conclusion

The ethyl acetate extract from flowers extracts of* Anthyllis henoniana* (Coss.) showed different levels of phytochemicals, antioxidant, and antipostprandial hyperglycemia activities. The highest content of phenolics plays a major role in determining the antioxidant and antihyperlipidemia activities of this plant. In conclusion, this study hands over a clear evidence for the ethnobotanical uses of* Anthyllis henoniana* (Coss.) flowers extract in the cure of diabetes mellitus. The encouraging results for such first antidiabetic effect study of* Anthyllis henoniana* lead us to enhance more specific investigations to isolate and identify the responsible bioactive molecule (s) in the futuristic research.

## Figures and Tables

**Table 1:** Phytochemical screening of *Anthyllis henoniana* flowers extracts.

	Hexane extract (HEx)	Ethyl acetate extract (EAEx)	Methanol extract (MEx)
Flavonoids	−	++	++
Alkaloids	−	−	−
Quinones	−	−	−
Sterols	++	+	−
Tropolones	−	−	−
Terpenoids	++	+	−
Tannins	−	+	+

(+) Weak presence; (++) presence; (−) absence.

**Table 2:** Antioxidant activity of *Anthyllis henoniana* flowers extracts using DPPH method and total antioxidant capacity (TAC).

Extracts	DPPH IC_50_ (mg/mL)	TAC mg of equivalents of Vitamin E/ g extract
HEx	-	6.13 ± 1.53
EAEx	2.34	50.61 ± 1.90
MEx	1.58	59.33 ± 1.66
Vitamin E	0.2	-

Values are expressed as the means ± SD of three independent assays (n=3).

**Table 3:** Correlation coefficients (r^*2*^values) of total polyphenol, flavonoid, and condensed tannins concentration, with DPPH activity expressed as IC_50_ values TAC expressed as mg of antioxidant /g of extract.

**Correlation**	***R*** ^2^
DPPH versus Polyphenols	0.722
DPPH versus Flavonoids	0.712
DPPH versus Tannins	0.710
TAC versus Polyphenols	0.679
TAC versus Flavonoids	0.420
TAC versus Tannins	0.685

**Table 4:** Measurement of body weights of different experimental groups of rats.

Groups	Day1	Day7	Day 14	Day 21	Day 28
Cont	179.56± 0.6	181.23 ± 0.12	185.23± 0.12	195.50 ± 1.23	204.12 ± 2.23
Diab	180.26 ± 1.23	178.12 ± 2.36^*∗∗∗*^	173.56 ± 1.12^*∗∗∗*^	173.66 ± 1.12^*∗∗∗*^	155.23± 1.20^*∗∗∗*^
Diab+EAEx	184.21 ± 2.2	188.56 ± 0.74^###@^	196.45 ± 0.12^###@@@^	205.33 ±3.74^###@@@^	210.56 ±0.04^###@@@^
Diab+Acar	185.4 ± 3.12^#^	189.23 ± 1.15^###^	195.23 ± 1.2^###^	200.45 ± 1.23^###^	203.16 ± 0.46

Values represent means ± SD (n = 8 for each group). Control groups (Cont) and diabetic control (Diab). Diab+ EAEx: diabetic rats treated with EAEx of* Anthyllis henoniana* at 400 mg/kg bw per day for 28 days; Diab+Acar: diabetic rats treated with acarbose at dose of 12 mg/kg bw per day for 28 days. The values are means ±SD (n=8 for each group). Values differ significantly at ^*∗∗∗*^p< 0.001, as considered significant compared to control groups;^#^p<0.05 and ^###^p < 0.001, as considered significant compared with diabetic rats;^@^p <0.05 and ^@@@^p < 0.001, as considered significant compared with diabetic rats treated with acarbose.

**Table 5:** OGTT levels of different experimental groups of rats.

Groups	0min	30min	60 min	120 min	240 min
Cont	96.3 ± 2.51	180.6 ± 3.21	130 ± 5	98 ± 2.6	82.3 ± 2.51
Diab	205 ± 1	245 ± 15	267 ± 12.5	258 ± 7.6	238 ± 7.4
Diab+EAEx	140 ± 5	170 ± 5	198 ± 2.64	155 ± 5	125 ± 5
Diab+Acar	106.3 ± 4.5	124 ± 3.21	125 ± 4.04	123 ±7.6	111.3 ± 3.21

Control group (Cont) and diabetic control (Diab). Diab+ EAEx: diabetic rats treated with EAEx of *Anthyllis henoniana *at 400 mg/kg bw per day for 28 days; Diab+Acar: diabetic rats treated with acarbose at dose of 12 mg/kg bw per day for 28 days. The values are means ±SD (n=8 for each group).

**Table 6:** Effect of ethyl acetate flowers extract of *Anthyllis henoniana* on lipid profile levels of experimental groups of rats.

Lipid profile	Control	Diab	Diab+ EAEx	Diab+Acar
T-Ch (mmol/L)	0.68 ±0.17	1.72 ±1.28^*∗∗∗*^	1.55 ± 0.58^*∗∗∗*#@@@^	0.60±0.17^*∗∗∗*###^
TG (mmol/L)	1.45±0.26	1.88 ±0.46^*∗∗∗*^	0.54 ±0.12^*∗∗∗*###@@@^	1.38±0.56^###^
LDL-Ch (mmol/L)	0.50 ±0.19	0.99 ± 0.08^*∗∗∗*^	0.6 ±0.23^###^	0.58 ± 0.28^*∗*###^
HDL-Ch (mmol/L)	0.65 ± 0.17	0.25 ± 0.08^*∗∗∗*^	0.55 ± 0.07^*∗∗*###@^	0.38 ± 0.84^*∗*#^

Values represent mean ± SD (n = 8 for each group). Control groups (Cont) and diabetic control (Diab). Diab+ EAEx: diabetic rats treated with EAEx of *Anthyllis henoniana *at 400 mg/kg bw per day for 28 days; Diab+Acar: diabetic rats treated with acarbose at dose of 12 mg/kg/bw per day for 28 days. Values differ significantly at^*∗*^p<0.05,^*∗∗*^p<0.01, and ^*∗∗∗*^p< 0.001, as considered significant compared to control groups;^#^p<0.05 and^ ###^p < 0.001, as considered significant compared with diabetic rats;^@^p <0.05 and ^@@@^p < 0.001, as considered significant compared with diabetic rats treated with acarbose.

**Table 7:** Plasma hepatic and renal parameters of experimental groups of rats.

Hepatic /Renal parameters	Cont	Diab	Diab+EAEx	Diab+Acar
AST (UI/L)	116.6± 1.20	178.25±5.63^*∗∗∗*^	125±5.15^###@@@^	151.66 ± 3.17^###@@@^
ALT (UI/L)	39.33±5.78	66.25± 1.18^*∗∗∗*^	34.66± 5.68^###@@@^	47.75± 6.00^###@@@^
Urea (mmol/L)	4.86 ± 8.57	8.99 ± 2.26^*∗∗∗*^	5.56±1.88^###^	7.00 ± 5.26^###^
Creatinine(umol/L)	18.54 ± 3.24	28.24 ± 3.17^*∗∗∗*^	19.13±4.5^###^	15.21 ± 5.25^###^

Values represent mean ± SD (n = 8 for each group). Control groups (Cont) and diabetic control (Diab). Diab+ EAEx: diabetic rats treated with EAEx of *Anthyllis henoniana *at 400 mg/kg bw per day for 28 days; Diab+Acar: diabetic rats treated with acarbose at dose of 12 mg/kg bw per day for 28 days. Values differ significantly at^*∗∗∗*^p< 0.001, as considered significant compared to control groups;^ ###^p < 0.001, as considered significant compared with diabetic rats;^@@@^p < 0.001, as considered significant compared with diabetic rats treated with acarbose.

**Table 8:** Effect of the ethyl acetate flowers extract of *Anthyllis henoniana* on oxidative stress markers of different experimental groups of rats.

Groups	SOD (U/mg protein)	CAT (umole H_2_O_2_/min/mg protein)	GSH (ug/mg protein)	TBARS (nmol MDA/mg protein)	AOPP (nmol/mg protein)
***Liver***					
Cont	70.12±1.30	1.70±1.32	40.12±1.25	11.00±0.25	0.16±1.25
Diab	60.01 ±1.20^*∗*^	0.80±0.02	12.3±0.02^*∗∗∗*^	28.50±1.21^*∗∗∗*^	0.67±1.05^*∗∗∗*^
Diab+ EAEx	64.55±7.39^#^	1.44±2.24^###^	27.07±1.9^###@@@^	16.38±9.36^###@@^	0.52±0.30^###^
Diab+ Acar	50.22± 1.39^###^	1.5±0.02^##^	30.00±0.4^###^	14.47±6.13^###^	0.32±0.02^###^
***Rein***					
Cont	36.59±5.70	1.25±0.44	17.85±3.97	20.12±1.03	0.11±1.56
Diab	18.24±1.70^*∗∗∗*^	0.11±0.82^*∗∗∗*^	9.06±1.19^*∗∗∗*^	24.00±0.23^*∗∗∗*^	0.30±1.231^*∗∗∗*^
Diab+ EAEx	30.16±0.03^###@@@^	0.40±0.83^##@^	13.12 ±5.06^###@^	19.81±5.38^###@^	0.15±0.08^###@@^
Diab+ Acar	20.36±2.36^###@@@^	0.79±0.12^###^	19.00±0.17^##^	18.31±1.50^###^	0.17±0.25^###^
***Pancreas***					
Cont	97.71±1.58	1.05±0.14	7.17±0.13	10.00±0.75	0.24±0.12
Diab	39.62±2.23^*∗∗∗*^	0.10±1.02^*∗∗∗*^	3.21±0.02^*∗∗∗*^	25.81±1.70^*∗∗∗*^	0.87±0.14^*∗∗∗*^
Diab+ EAEx	53.63±1.06^###@@@^	2.89±1.12^###^	8.50±6.10^###^	10.37±3.19^###^	0.33±0.13^###^
Diab+Acar	65.15±4.92^###^	0.81±1.12^###^	2.53±1.00^###^	12.71±1.73^###^	0.25±0.23^###@@@^

Values represent means ± SD (n = 8 for each group). Control groups (Cont) and diabetic control (Diab). Diab+ EAEx: diabetic rats treated with EAEx of *Anthyllis henoniana *at 400 mg/kg bw per day for 28 days; Diab+Acar: diabetic rats treated with acarbose at dose of 12 mg/kg bw per day for 28 days. Values differ significantly at^*∗*^p<0.05 and ^*∗∗∗*^p < 0.001, as considered significant compared to control groups;^#^p<0.05^##^ and p<0.01,^###^p < 0.001, as considered significant compared with diabetic rats; ^@^p<0.05, ^@@^p<0.01, and ^@@@^p < 0.001, as considered significant compared with diabetic rats treated with acarbose.

## Data Availability

All data used to support the findings of this study are available from the corresponding author upon a reasonable request.

## References

[1] Zimmet P., Alberti K. G. M. M., Shaw J. (2001). Global and societal implications of the diabetes epidemic. *Nature*.

[2] Rossetti L., Giaccari A., DeFronzo R. A. (1990). Glucose toxicity. *Diabetes Care*.

[3] Chiasson J.-L., Rabasa-Lhoret R. (2004). Prevention of type 2 diabetes: insulin resistance and *β*-cell function. *Diabetes*.

[4] Eichler H. G., Korn A., Gasic S., Pirson W., Businger J. (1984). The effect of a new specific *α*-amylase inhibitor on post-prandial glucose and insulin excursions in normal subjects and Type 2 (non-insulin-dependent) diabetic patients. *Diabetologia*.

[5] Landrault N., Poucheret P., Azay J. (2003). Effect of a polyphenols-enriched chardonnay white wine in diabetic rats. *Journal of Agricultural and Food Chemistry*.

[6] Chidambara Murthy K. N., Singh R. P., Jayaprakasha G. K. (2002). Antioxidant activities of grape (Vitis vinifera) pomace extracts. *Journal of Agricultural and Food Chemistry*.

[7] O'Keefe J. H., Bell D. S. (2007). Postprandial hyperglycemia/hyperlipidemia (postprandial dysmetabolism) is a cardiovascular risk factor. *American Journal of Cardiology*.

[8] Kim J., Kwon C., Son K. H. (2000). Inhibition of alpha-glucosidase and amylase by luteolin, a flavonoid. *Bioscience, Biotechnology, and Biochemistry*.

[9] Lee D.-S., Lee S.-H. (2001). Genistein, a soy isoflavone, is a potent *α*-glucosidase inhibitor. *FEBS Letters*.

[10] Passamonti S., Vrhovsek U., Vanzo A., Mattivi F. (2005). Fast access of some grape pigments to the brain. *Journal of Agricultural and Food Chemistry*.

[11] Bischoff H., Puls W., Krause H. P., Schutt H., Thomas G. (1985). Pharmacological properties of novel glucosidase inhibitors BAY m 1099 (miglitol) and BAY o 1248. *Diabetes Research and Clinical Practice*.

[12] Oboh G., Isaac A. T., Akinyemi A. J., Ajani R. A. (2014). Inhibition of key enzymes linked to type 2 diabetes and sodium nitroprusside induced lipid peroxidation in rats’ pancreas by phenolic extracts of avocado pear leaves and fruit. *International Journal of Biomedical Science*.

[13] Unnikrishnan P. S., Suthindhiran K., Jayasri M. A. (2014). Inhibitory potential of *Turbinaria ornata* against key metabolic enzymes linked to diabetes. *BioMed Research International*.

[14] Nain P., Saini V., Sharma S., Nain J. (2012). Antidiabetic and antioxidant potential of Emblica officinalis Gaertn. leaves extract in streptozotocin-induced type-2 diabetes mellitus (T2DM) rats. *Journal of Ethnopharmacology*.

[15] Saleh P., Asghari B., Esmaeil M. A., Dehghan H., Ghazi I. (2013). *α*-glucosidase and *α*-amylase inhibitory effect and antioxidant activity of ten plant extracts traditionally used in Iran for diabetes. *Journal of Medicinal Plants Research*.

[16] Derbel S., Chaieb M. (2013). Growth establishment and phenology of four woody Saharan species. *African Journal of Ecology*.

[17] Aronson J., Floret C., Le Floc'h E., Ovalle C., Pontanier R. (1993). Restoration and rehabilitation of degraded ecosystems in arid and semi‐arid lands. II. Case studies in Southern Tunisia, Central Chile and Northern Cameroon. *Restoration Ecology*.

[18] Harborne J. B. (1973). *Phytochemical Methods*.

[19] Singleton V. L., Rossi J. A. (1965). Colorimetry of total phenolics with phosphomolybdic-phosphotungstic acid reagents. *American Journal of Enology and Viticulture*.

[20] Zhishen J., Mengcheng T., Jianming W. (1999). The determination of flavonoid contents in mulberry and their scavenging effects on superoxide radicals. *Food Chemistry*.

[21] Price M. L., Scoyoc S. V., Butler L. G. (1978). A critical evaluation of the vanillin reaction as an assay for tannin in sorghum grain. *Journal of Agricultural and Food Chemistry*.

[22] Ak T., Gülçin I. (2008). Antioxidant and radical scavenging properties of curcumin. *Chemico-Biological Interactions*.

[23] Oyaizu M. (1986). Studies on products of browning reaction: antioxidative activity of products of browning reaction prepared from glucosamine. *The Japanese Journal of Nutrition and Dietetics*.

[24] Prieto P., Pineda M., Aguilar M. (1999). Spectrophotometric quantitation of antioxidant capacity through the formation of a phosphomolybdenum complex: specific application to the determination of vitamin E. *Analytical Biochemistry*.

[25] Wang H., Du Y.-J., Song H.-C. (2010). *α*-Glucosidase and *α*-amylase inhibitory activities of guava leaves. *Food Chemistry*.

[26] Nishioka T., Kawabata J., Aoyama Y. (1998). Baicalein, an *α*-glucosidase inhibitor from *Scutellaria baicalensis*. *Journal of Natural Products*.

[27] Ranilla L. G., Kwon Y.-I., Apostolidis E., Shetty K. (2010). Phenolic compounds, antioxidant activity and *in vitro* inhibitory potential against key enzymes relevant for hyperglycemia and hypertension of commonly used medicinal plants, herbs and spices in Latin America. *Bioresource Technology*.

[28] Mustafa K. G., Ganai B. A., Akbar S., Dar M. Y., Masood A. (2012). *β*-Cell protective efficacy, hypoglycemic and hypolipidemic effects of extracts of *Achillea millifolium* in diabetic rats. *Chinese Journal of Natural Medicines*.

[29] Zanatta L., de Sousa E., Cazarolli L. H. (2007). Effect of crude extract and fractions from Vitex megapotamica leaves on hyperglycemia in alloxan-diabetic rats. *Journal of Ethnopharmacology*.

[30] Ben Abdallah Kolsi R., Ben Gara A., Jardak N. (2015). Inhibitory effects of Cymodocea nodosa sulphated polysaccharide on *α* -amylase activity, liver-kidney toxicities and lipid profile disorders in diabetic rats. *Archives of Physiology and Biochemistry*.

[31] Friedewald W. T., Levy R. I., Fredrickson D. S. (1972). Estimation of the concentration of low-density lipoprotein cholesterol in plasma, without use of the preparative ultracentrifuge. *Clinical Chemistry*.

[32] Yoshioka T., Kawada K., Shimada T., Mori M. (1979). Lipid peroxidation in maternal and cord blood and protective mechanism against activated-oxygen toxicity in the blood. *American Journal of Obstetrics & Gynecology*.

[33] Kayali R., Çakatay U., Akçay T., Altuğ T. (2006). Effect of alpha-lipoic acid supplementation on markers of protein oxidation in post-mitotic tissues of ageing rat. *Cell Biochemistry & Function*.

[34] Marklund S., Marklund G. (1974). Involvement of the superoxide anion radical in the autoxidation of pyrogallol and a convenient assay for superoxide dismutase. *European Journal of Biochemistry*.

[35] Paglia D. E., Valentine W. N. (1967). Studies on the quantitative and qualitative characterization of erythrocyte glutathione peroxidase. *The Journal of Laboratory and Clinical Medicine*.

[36] Aebi H. (1984). Catalase in vitro. *Methods in Enzymology*.

[37] Lowry O. H., Rosebrough N. J., Farr A. L., Randall R. J. (1985). Protein measurement with the Folin phenol reagent. *Journal of Biological Chemistry*.

[38] Chung I.-M., Kim E.-H., Yeo M.-A., Kim S.-J., Seo M.-C., Moon H.-I. (2011). Antidiabetic effects of three Korean sorghum phenolic extracts in normal and streptozotocin-induced diabetic rats. *Food Research International*.

[39] El Hadi M. A. M., Zhang F.-J., Wu F.-F., Zhou C.-H., Tao J. (2013). Advances in fruit aroma volatile research. *Molecules*.

[40] Naczk M., Shahidi F. (2004). Extraction and analysis of phenolics in food. *Journal of Chromatography A*.

[41] Krishnaiah D., Bono A., Sarbatly R., Anisuzzaman S. M. (2015). Antioxidant activity and total phenolic content of an isolated Morinda citrifolia L. methanolic extract from Poly-ethersulphone (PES) membrane separator. *Journal of King Saud University - Engineering Sciences*.

[42] Sharififar F., Dehghn-Nudeh G., Mirtajaldini M. (2009). Major flavonoids with antioxidant activity from *Teucrium polium* L.. *Food Chemistry*.

[43] Miceli N., Buongiorno L. P., Celi M. G. (2015). Role of the flavonoid-rich fraction in the antioxidant and cytotoxic activities of. *Natural Product Research*.

[44] Gardeli C., Vassiliki P., Athanasios M., Kibouris T., Komaitis M. (2008). Essential oil composition of Pistacia lentiscus L. and Myrtus communis L.: Evaluation of antioxidant capacity of methanolic extracts. *Food Chemistry*.

[45] Rice-Evans C. A., Miller N. J., Paganga G. (1997). Antioxidant properties of phenolic compounds. *Trends in Plant Science*.

[46] Kaewamatawong R. (2008). Secondary metabolites and pharmacological activities of *Bauhinia* genus plants. *Science journal Ubon Ratchathani University: ScJ ubu*.

[47] Gao T., Yao H., Song J. (2010). Identification of medicinal plants in the family Fabaceae using a potential DNA barcode ITS2. *Journal of Ethnopharmacology*.

[48] Sireesha Y., Kasetti R. B., Nabi S. A., Swapna S., Apparao C. (2011). Antihyperglycemic and hypolipidemic activities of Setaria italica seeds in STZ diabetic rats. *Pathophysiology*.

[49] Fontana Pereira D., Cazarolli L. H., Lavado C. (2011). Effects of flavonoids on *α*-glucosidase activity: Potential targets for glucose homeostasis. *Nutrition Journal *.

[50] Cazarolli L. H., Zanatta L., Alberton E. H. (2008). Flavonoids: cellular and molecular mechanism of action in glucose homeostasis. *Mini-Reviews in Medicinal Chemistry*.

[51] Tadera K., Minami Y., Takamatsu K., Matsuoka T. (2006). Inhibition of *α*-glucosidase and *α*-amylase by flavonoids. *Journal of Nutritional Science and Vitaminology*.

[52] Kurakane S., Yamada N., Sato H., Igarashi K. (2011). Anti-diabetic effects of *Actinidia arguta* polyphenols on rats and KK-Ay mice. *Food Science and Technology Research*.

[53] Park H. Y., Toume K., Arai M. A., Koyano T., Kowithayakorn T., Ishibashi M. (2014). *β*-Sitosterol and flavonoids isolated from Bauhinia malabarica found during screening for Wnt signaling inhibitory activity. *Journal of Natural Medicines*.

[54] Fumelli P., Romagnoli F., Carlino G., Fumelli C., Boemi M. (1996). Diabetes mellitus and chronic heart failure. *Archives of Gerontology and Geriatrics*.

[55] Assmann G., Nofer J.-R. (2003). Atheroprotective effects of high-density lipoproteins. *Annual Review of Medicine*.

[56] Shah P. K., Kaul S., Nilsson J., Cercek B. (2001). Exploiting the vascular protective effects of high-density lipoprotein and its apolipoproteins: An idea whose time for testing is coming, part I. *Circulation*.

[57] Young I. S. (2005). Lipids for psychiatrists - An overview. *Journal of Psychopharmacology*.

[58] Daisy P., Rajathi M. (2009). Hypoglycemic effects of Clitoria ternatea Linn. (Fabaceae) in alloxan-induced diabetes in rats. *Tropical Journal of Pharmaceutical Research*.

[59] Achliya G. S., Wadodkar S. G., Dorle A. K. (2004). Evaluation of hepatoprotective effect of Amalkadi Ghrita against carbon tetrachloride-induced hepatic damage in rats. *Journal of Ethnopharmacology*.

[60] Thabrew M. I., Joice P. D. T. M., Rajatissa W. (1987). A comparative study of the efficacy of Pavetta indica and Osbeckia octandra in the treatment of liver dysfunction. *Planta Medica*.

[61] Fortson W. C., Tedesco F. J., Starnes E. C., Shaw C. T. (1985). Marked elevation of serum transaminase activity associated with extrahepatic biliary tract disease. *Journal of Clinical Gastroenterology*.

[62] Hultcrantz R., Glaumann H., Lindberg G., Nilsson L. (1986). Liver investigation in 149 asymptomatic patients with moderately elevated activities of serum: Aminotransferases. *Scandinavian Journal of Gastroenterology*.

